# Emergency nurses’ disaster preparedness competencies: A focused mapping review and synthesis

**DOI:** 10.4102/hsag.v30i0.2770

**Published:** 2025-01-31

**Authors:** Tshepo L. Motsepe, Shelley Schmollgruber

**Affiliations:** 1 Department of Nursing Education, Faculty of Health Sciences, University of the Witwatersrand, Johannesburg, South Africa

**Keywords:** disaster preparedness, emergency nurses, competencies, emergency nurses’ disaster competencies, disaster management

## Abstract

**Background:**

Emergency nurses are at the front line of a health institution and are deemed competent to manage various situations, with disaster being one of them. Preparing for disaster is crucial as it will eliminate delays in providing care in conditions that would be abnormal in the daily running of an emergency department.

**Aim:**

To identify emergency nurses’ disaster preparedness competencies in caring for patients during a disaster.

**Setting:**

The study was conducted in emergency departments and trauma centres that respond to disasters and mass casualty incidents.

**Methods:**

A focused mapping review and synthesis was conducted where, articles published between 2006 and 2022 were searched utilising keywords relating to emergency nurses’ disaster preparedness through the following databases: SCOPUS, Sabinet, EBSCO host, ProQuest, PubMed and Google Scholar. Data were extracted from articles that met the inclusion criteria and placed into an extraction table where themes were developed.

**Results:**

Of the 38 articles identified, duplicates were removed and 15 remained after an abstract review and full-text download. The review yielded five themes: communication, preparation and planning, incident management system, safety and security and intervention, the most dominant disaster competencies.

**Conclusion:**

Communication, incident management, safety and security, preparedness and planning are the most identified integral domains within other identified domains. Integrating these aspects in nurses’ continuous training and curricula is recommended to emphasise and enhance disaster preparedness and management knowledge.

**Contribution:**

This review has emphasised emergency nurses’ disaster preparedness domains, enhancing disaster preparedness and management knowledge and further influencing education and training for undergraduate and postgraduate nursing students.

## Introduction

Disasters of various magnitudes occur globally, affecting the community, the health system and the provision of immediate care. Thus, with such occurrences, emergency nurses are expected to be prepared and manage such an occurrence diligently.

The World Health Organization (WHO) defines a disaster as ‘an occurrence that disrupts the normal conditions of existence and causes a level of suffering that exceeds the capacity to adjust the affected community’ (WHO 2008). Emergency nurses are at the forefront of healthcare provision in a hospital setting, and they are expected to be competent in their nursing management in such an acute area, including disaster management.

The WHO defines competency as ‘the ability of the nurse to integrate and apply the knowledge, skill, judgement and personal attributes required to practice safely and ethically in a designated role in setting’ (WHO 2008:27). The International Council of Nurses (ICN) further described competence in the health professions as ‘the knowledge that enables a practitioner to perform activities consistently in a safe manner’ (WHO & ICN 2009:34). To ensure competency in responding to disasters, the ICN with WHO released the disaster nursing competencies framework for the generalist nurse to clarify roles the nurse needs to assume in a disaster and assist in the development of disaster training and education.

These organisations further elaborated on the importance of having a standard set of competencies in disaster nursing, enabling nurses to work globally in various settings (WHO & ICN 2009). Based on the 2009 ‘ICN framework of disaster nursing competencies’, these competencies were revised and published in 2019 as ‘Core Competencies in Disaster Nursing: Competencies for Nurses Involved in Emergency Medical Teams (Level III)’. This updated version (version 2.0) outlined what nurses should know and be able to do during disaster management, as version 1.1 focused more on generalist nurses and did not include advanced and specialist nurses (ICN 2019).

Eight new domains were introduced in version 2.0 compared to the 10 of those of 2009, and three levels of nurses were included as per ICN description in version two of the competencies.

With the WHO and ICN introducing disaster nursing competencies, Marin and Witt (2015) identified healthcare nurses’ competencies when responding to hydrological disasters in rural areas, with 30 competencies being identified and classified into eight domains.

Chegini et al. (2022) evaluated the levels of disaster core competencies and emergency department nurses’ preparedness. Gaps within disaster preparedness and core competencies of emergency nurses were identified. The study extrapolated that emergency nurses had inadequate disaster core competency and were moderately prepared for disasters (Chegini et al. 2022). As gaps in disaster preparedness and competencies are identified through various reviews, this review (A Focused Mapping Review and Synthesis) aimed to identify and map emergency nurses’ disaster preparedness competencies.

## Methods

A Focused Mapping Review and Synthesis (FMRS) was conducted to identify emergency nurses’ disaster preparedness competencies. According to James, Randall and Haddaway (2016), a mapping review is a review method that ‘collates, describes and catalogues available evidence relating to a topic or question of interest’, it provides ‘an overview of a research area and pinpoints knowledge gaps that might require “more” complete systematic reviews or further primary search’ (Booth 2016:14).

The steps identified in the FMRS by Bradbury-Jones et al. (2019) are as follows: (1) focus, (2) mapping and (3) synthesis. Journals were purposively selected to answer the review question, ‘What are emergency nurses’ disaster competencies?’

### Focus

The focus phase is the foundational stage of a FMRS, where two areas, the timeframe for included studies and the sources of information to be explored, are defined (Bradbury-Jones et al. 2019). This process-orientated step provides a panoramic view from the multiple sources obtained (Bradbury-Jones et al. 2019). Thus, the FMRS was conducted between September 2022 and February 2023, with the last search done in March 2023, granting the researchers sufficient time to obtain articles to answer the review question. ‘Emergency nurses disaster competencies, disaster nursing, disaster nursing competencies and “disaster nurses” preparedness’ were search words utilised to search for articles relevant to the review. Articles were obtained from hand-searched journals, reference list checks and electronic databases such as SCOPUS, Sabinet, EBSCO host, ProQuest, PubMed and Google Scholar. These electronic databases were utilised as they offer a wide range and access to articles, full-text coverage and broader coverage of nursing literature, abstracts and unpublished literature. Researchers screened titles and abstracts from the retrieved studies independently for eligibility against the inclusion criteria. From that, a full-text download was done, and the researchers reached a consensus on the final articles to be included in the review.

According to Bradbury-Jones et al. (2019), this also introduces a modest-quality assessment level. Thus, high-ranking journals were included as identified in Table 1 to obtain the best comprehensive and up-to-date knowledge utilising Scimango Journal and country ranking in the fields ‘nursing’, ‘emergency nursing’, ‘all countries’ and ‘all journals’.

**TABLE 1:** Scimango journal rankings.

Number	Journal
1	Prehospital and Disaster Medicine
2	Annals of Emergency Medicine
3	Collegian
4	Sage Open Nursing
5	International Emergency Nursing
6	PLOS One
7	Nurse Education in Practice
8	Nurse Education Today
9	BMC Medical Education
10	Iranian Journal of Public Health
11	Nurse Education Today
12	International Nursing Review
13	Trauma Monthly
14	Rural and Remote Health
15	Chinese Nursing Research

**FIGURE 1:**
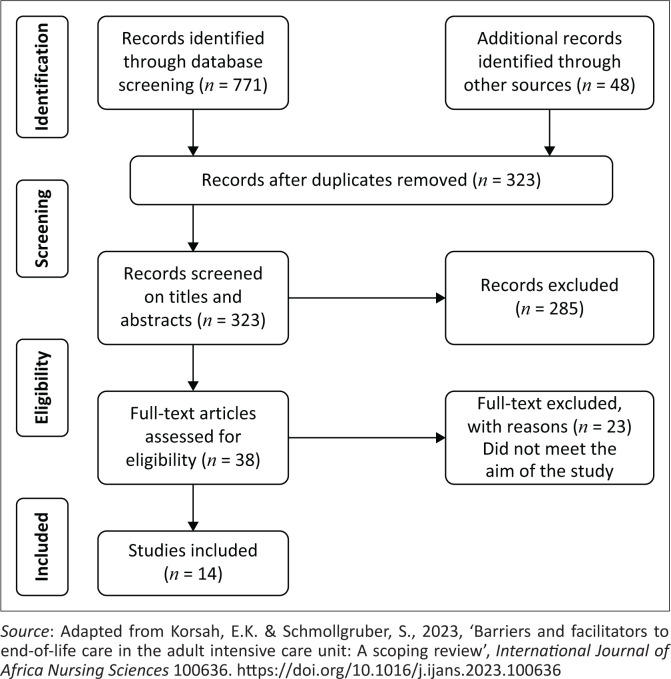
PRISMA flow diagram.

From the process, the search yielded 771 articles. Following inclusion and exclusion criteria, screening for relevance and eligibility and abstracts and titles were undertaken for articles and titles. Screening was done independently by both the researcher and the supervisor, who later collaborated to ensure consensus and rigour for article inclusion. A third independent reviewer was included to resolve conflict, and further discussions were conducted to ensure methodological validity. A full-text download for articles that met the inclusion criteria was included, and the articles were read in full to gain a deeper understanding of the abstract reviewed. Furthermore, articles were compared and agreed upon from the retrieved articles by the researchers, of which 38 articles were retrieved, and following the inclusion criteria, 15 articles were included for the FMRS, as indicated in the PRISMA diagram (Figure 1).

### Mapping

Bradbury-Jones et al. (2019:457) describe mapping as identifying and visually presenting contours and limits within a specific body of literature. Similarly, Grant and Booth (2009) view mapping as creating systematic representations to organise and characterise studies. For this review, 15 articles that focused on nursing disaster competencies were identified, with the majority referring to the 2009 ICN Framework of Disaster Nursing Competencies and the 2019 ICN Core Competencies in Disaster Nursing, Version 2.0, which were additionally included in the results.

The mapping stage included calibration, whereby the ‘need for frequent points of contact and deliberation among the review team in agreeing to the parameters of the review’ (Bradbury-Jones et al. 2019:455), the researcher and supervisor agreed on the definitions of the terms ‘disaster’ and ‘competencies’ and how they are utilised within the review to align with the context of the study.

### Synthesis

This stage involves analysing the identified literature by identifying gaps and providing an understanding of the current state of knowledge (Bradbury-Jones et al. 2019). Thus, results were extracted and presented on a data extraction table (Table 2) with the following headings: author and year, journal, title or aim or objective of the study and key findings or results. Braun and Clarke’s (2006) thematic analysis principles were utilised to group findings into codes, categories and themes. The researcher and supervisor independently read and re-read each article to familiarise themselves with the results. Themes were subsequently generated and reported in a narrative format under each theme.

**TABLE 2:** Data extraction table.

No.	Author and year	Journal	Title or aims or objectives	Key findings or results
1	Marin and Witt (2015)	Prehospital and Disaster Medicine	To identify hospital nurses’ competencies in disaster situations	**Seventeen competencies were validated, with the following domains:** Management Healthcare Communication and Education
2	Schultz et al. (2012)	Annals of Emergency Medicine	To identify a set of core competencies and performance objectives based on the knowledge, skills and attitudes required by the specific target audience (emergency department nurses, emergency physicians and out-of-hospital emergency medical services personnel) to ensure they can treat the injuries and illnesses experienced by victims of disasters regardless of cause	**Nineteen domains indicated:** Nomenclature IMS (NRF, NIMS, ICS) Recognition, notification, initiation and data collection Communication (inter/intra-agency and media) Resource management Volunteer management (invited, spontaneous or convergent) GO- and NGO-sponsored response teams Public health and safety Patient triage Surge capacity or capability Patient identification and tracking Transportation Decontamination Clinical considerations Special-needs populations Evacuation Critical thinking or situational awareness Ethical principles and challenges Psychosocial issues
3	Satoh et al. (2018)	Sage Open Nursing	To identify the disaster nursing knowledge and competencies among university nursing students who participated in relief activities following the 2016 Kumamoto earthquakes.	Understanding and implementation of assistance to victims in collaboration with other disaster response team members Understanding of natural disasters’ influence on victims Ethical practice in a disaster recovery area Understanding of their role within the disaster relief organisation
4	Murphy et al. (2019)	International Emergency Nursing	To identify essential disaster medicine competencies for emergency department registered nurses through expert consensus	**Twelve competencies:** Detection of and response to an event The incident command system Ethical issues in triage Epidemics and surveillance Biological Isolation or quarantine Decontamination Communication Psychological Special populations Accessing critical resources Overall familiarity with disaster preparedness
5	Karnjuš, Prosen and Ličen (2021)	PLOS One	To explore how registered nurses perceive the core competencies entailed in disaster nursing, their role in disaster management and the potential barriers to developing disaster nursing in Slovenia	**Competencies: ‘Nurses’ core competencies in disaster management:** Demonstrate a capability of following and working in an incident management system. Identify potential threats with medical implications in response areas. Provide defensible resolutions to ethical dilemmas that arise in disasters Recognise the objective of the disaster plan Explain the principles of crisis communication in crisis intervention and risk management Maintain disaster knowledge in areas relating to disaster and disaster nursing Explain nurses’ roles in disaster areas (e.g. shelters, emergency care sites) Engage in the planning of meeting healthcare needs in a disaster Identify the disaster plans at the time of a disaster in the workplace Describe ethical strategies for allocating scarce resources to maximise population results during triage and management Name the relevant steps for requesting psychological first aid for responders, patients and other victims Understand how to prioritise care and manage multiple situations Resources and supplies should be managed to provide care in the community To reduce risk, vulnerable populations should be identified vulnerable populations and activities should be accurately coordinated Be involved in the creation of new nursing disaster guidelines Explain the disaster management phases: prevention/mitigation, preparedness, response and recovery or rehabilitation The disaster response team should be offered up-to-date information regarding healthcare issues and resource needs Create and sustain a family and personal preparedness plan Demonstrate understanding of the disaster plan component To ensure continuity of patient information, recordkeeping processes should be adhered to Name and apply principles for managing patients with the most common victim presentations, e.g. environmental illnesses, burns, blast and crush injuries; nuclear, biological and chemical Ensure safe and effective patient transportation during a disaster Participate in workplace and community drills Take part in securing adequate personnel, supplies, equipment and space for patient care (surge capacity) Identify and communicate important information immediately to appropriate authorities Understand relevant disaster terminology Prioritise patients to maximise survivability
6	Alfred et al. (2015)	Nurse Education in Practice	Part 1: An educational journey towards disaster nursing competencies: A curriculum in action provides an overview of the curricular tools used to ensure adequate coverage of disaster nursing concepts across the curriculum	**Prevention and/or mitigation competencies** Risk reduction, disease prevention and health promotion Policy development and planning **Preparedness competencies** Ethical practice, legal practice and accountability Communication and information sharing Education and preparedness **Response competencies** Care of the community Care of individuals and families Psychological care Care of vulnerable populations **Recovery or rehabilitation competencies** Long-term individual, family and community recovery
7	Alim, Kawabata and Nakazawa (2015)	Nurse Education Today	To evaluate the effectiveness of a disaster preparedness training programme followed by a disaster drill designed for nursing students	Topics for training: **Topic 1: Introduction to disaster preparedness for nurses** A general description of health problems in disaster The role of nurses in every phase of a disaster Nursing care principles in a disaster **Topic II: Command and management for healthcare** Incident command system Multiagency coordinating systems Public information systems Public information systems **Topic III: Basic life support treatment in disaster 1** Triage methods in the field Cardio-pulmonary resuscitation **Topic IV: basic life support treatment in disaster 2** Management of head trauma, chest injury, bleeding and fracture
8	Hsu et al. (2006)	BMC Medical Education	Apply systematic evidence-based consensus-building methods to derive educational competencies and objectives in criteria-based preparedness and response relevant to all hospital healthcare workers	**Cross-cutting competencies for healthcare workers:** Recognise a potentially critical event and implement initial actions Apply the principles of critical event management Demonstrate critical event safety principles Understand the institutional emergency operations plan Demonstrate effective essential communications of event Understand the incident command system and your role in it Demonstrate the knowledge and skills needed to fulfil your role during a critical event
9	Taskiran and Baykal (2019)	International Review	To identify nurses’ perceptions of their disaster preparedness and core competencies	Critical thinking skills Special diagnostic skills General diagnostic skills Technical skills Communication skills
10	Al Thobaity, Plummer and Williams (2017)	International Emergency Nursing	To identify the most common domains of the core competencies of disaster nursing	**Common domains:** Communication Planning Decontamination and safety Incident command system Ethics
11	Hutton, Veenema and Gebbie (2016)	Prehospital Disaster Medicine	To review the use of the ICN framework of disaster nursing competencies.	The majority of respondents indicated making use of the ICN framework of disaster nursing competencies Competencies were held in high esteem and valued by various organisations as the cornerstone of their disaster education, and they were also used for the continued professional development of disaster nursing Indication for inclusion of psychosocial elements of nurses caring for themselves and their colleagues
12	Nejadshafiee et al. (2020)	Trauma Monthly	To assess hospital nurses’ disaster competencies in such situations	**Competencies:** Management Ethical Personal Technical
13	Menegat and Witt (2018)	Rural and Remote Health	To identify primary healthcare nurses’ competencies when responding to hydrological disasters in rural areas	**Competencies:** Leadership and management Teamwork Healthcare Community orientated Communication Psychological care Health Surveillance Educational
14	Li, Li and Xu (2015)	Chinese Nursing Research	Defines the concepts and elements of disaster nursing, including disaster nursing skill requirements and architectural framework	**Competencies:** Competency in preparedness for the prevention of disasters Emergency rescue competency The competence of rational allocation and management of resources

*Source:* Adapted from Korsah, E.K. & Schmollgruber, S., 2023, ‘Barriers and facilitators to end-of-life care in the adult intensive care unit: A scoping review’, *International Journal of Africa Nursing Sciences* 100636. https://doi.org/10.1016/j.ijans.2023.100636

Note: Please see the full reference list of this article, https://doi.org/10.4102/hsag.v30i0.2770 for more information.

IMS, Incident Management System; NRF, National Response Framework; NIMS, National Incident Management System; ICS, Incident Command System; ICN, Internation Council of Nurses; GO, governmental organizations; NGO, nongovernmental organization.

### Ethical considerations

Ethical clearance to conduct this study was obtained from the University of the Witwatersrand Human Research Ethics Committee (reference no.: M190983).

## Results

The extracted data were interpreted utilising thematic analysis, which yielded five themes: communication, preparation and planning, incident management system, safety and security and intervention as the most dominant disaster competencies within those identified.

### Communication

Communication is essential in a disaster as it mitigates detriments from occurring during a disaster. Thus, emergency nurses should learn critical event communication skills for an effective disaster response. From the competencies developed by Hsu et al. (2006), two objectives within the communication competency, communication overview and implementation, were identified. These authors elaborated on these objectives as the basis of standardised training with six other competencies and objectives. For a healthcare worker to be competent within the communication overview, one must identify the appropriate communication steps, which information should be reported, to which authority should reporting be done and the alternative modalities (Hsu et al. 2006).

With the aim of ‘identifying a set core competencies and performance objectives based on knowledge, skills and attitudes required by the specific target audience’ (emergency department nurses, emergency physicians and out-of-hospital emergency medical services personnel), Schultz et al. (2012:196) developed a framework of 19 content categories (domains), 19 core competencies and more than 90 performance objectives for acute medical personnel to provide them with an effective all-hazard disaster response. From these core competencies, a domain of detection and communication was identified and indicated within the education framework.

Identified by the ICN as a domain, communication is further explained as an ‘approach of conveying and updating essential information within one’s place of work or emergency assignment and documenting actions taken, and decisions made’ (ICN 2022:8), indicating the importance of the domain in disaster planning.

### Preparation and planning

A framework of 19 domains, 19 competencies and 93 performance objectives were identified for all health professionals in a disaster to address the requirements of an effective all-hazards disaster response by Schultz et al. (2012). From these competencies, the domain, preparation and planning were identified, which focused on demonstration proficiency of both the use of an all-hazard framework and addressing health-related needs, values and perspectives of all ages and populations in regional, community and institutional disaster planning (Schultz et al. 2012). Supported by the South African Nursing Council (SANC), the preparatory phase was identified as a phase whereby emergency nurses are expected to display competencies related to preparedness and apply health policy and organisational and personnel planning for emergencies (SANC 2019).

Furthermore, the ICN explains this domain as whereby actions are taken to increase readiness and confidence in activities to be taken during an emergency (ICN 2022). Murphy et al. (2019) identified that should a disaster occur, preparedness aims to improve preventive, response and preparation actions to manage such an occurrence. It is further mentioned that the preparation is based on various factors in disaster planning, such as institutional development of scientific and technological support structures, including human resources, monitoring, cultural changes, epidemiological studies on disaster and contingency planning (Murphy et al. 2019).

### Incident management system

Various structures and plans are developed and implemented for practical Incident Management Systems (IMS). The ICN explains incident management as a domain where ‘structures of disasters or emergency response are required by various countries with action that make them effective and efficient’ (ICN 2002:08). Murphy et al. (2019) identified 10 competencies under incident command systems whereby IMS planning was elaborated under various aspects including having a content of the emergency operational plans in one’s organisations and the ability of an institution preparedness level for responding to a large-scale disaster event.

Furthermore, Hsu et al. (2006) developed seven competencies with 21 objectives for critical event training for healthcare workers. From the competencies, an incident management system was identified.

### Safety and security

Safe practices and self-protection by the healthcare team can mitigate further disasters from occurring during a disaster occurrence. Murphy et al. (2019) describe safety and security as the ability of healthcare professionals to demonstrate proficiency in preventing and mitigating health, safety and security risks to themselves and others in a disaster or public health emergency. Additionally, to use the proper personal protective equipment at a disaster scene or a receiving facility and finally to show proficiency in victim decontamination at a disaster scene or receiving facility (Hsu et al. 2006). The ICN identified safety and security as a domain where competency is shown in that safe practices are practised by nurses, their colleagues and patients to ensure there is no addition to the burden of response through unsafe practices (ICN 2022).

### Interventions

After an assessment, interventions are done based on the assessment findings. Thus, in a disaster occurrence, interventions are implemented in response to an assessment of patients or families or communities within the incident management of the disaster event (ICN 2022). Schultz et al. (2012) included the domain of clinical or public health assessment and interventions, including competencies related to the domain. With the three levels of nurses explained by ICN, each nurse has roles; thus, within the domain of intervention, all nurses have specific interventions they are to encompass. Murphy et al. (2019) also outlined the competencies under the detection of and response to an event. In South Africa, the SANC has outlined that an emergency nurse specialist is expected to display competencies within major incident and disaster practice concerning preparedness and organisation and to have the skill to implement appropriate responses within an ethically challenging environment, simultaneously applying health policy and organisational and personnel planning for emergencies (SANC 2019:10).

## Discussion

The review identified various reported emergency nurse’s disaster competencies. These competencies include diverse aspects such as disaster guidelines and plans, disaster nursing roles, communication and aspects thereof, coordination of a nursing team during a disaster, safety, triage protocols, physical assessment and nursing intervention. Thus, five themes emerged from the review: Planning and preparedness, communication, incident management system, safety and security and interventions. Redness influences preparedness and care for disaster.

A study conducted by Marin and Witt (2015) identified 17 competencies that were organised following the WHO emergency management phases, with areas of management, healthcare, communication and education as the identified domains. Furthermore, as competencies were elaborated, disaster preparedness focused on the presence and formulation of emergency operations plans with contingency planning and disaster education and training also identified (Marin & Witt 2015). However, participants in Karnjuš et al. (2021) explained that there are barriers to developing core competencies, such as a lack of disaster nursing experts and workplace training programmes.

Nejadshafiee et al. (2020) conducted a cross-sectional study to assess hospital nurses’ disaster competencies using a self-report questionnaire. Nejadshafiee et al.’s (2020) results identified four competency domains: management, ethics, personal and technical aspects similar to a few recognised by Murphy et al. (2019), such as ethics. Thus, Nejadshafiee et al. (2020) concluded their study by identifying that management and technical competencies were moderate and with good ethical personal competencies. However, between rounds, some experts in Murphy et al. (2019) expressed concerns that specific competencies, such as ‘symptoms for varying injury mechanisms including chemical, explosive and shootings’ and ‘principles of treatment and care’ are not disaster medicine-specific competencies (Murphy et al. 2019).

Implementing safe and timely interventions during a disaster is essential, thus requiring nurses to be aware of their roles during a disaster. Incorporating disaster preparedness training into emergency nurses’ curriculum, formulating disaster preparedness, disaster management plans and nurses’ roles have been identified as an action that needs to be undertaken by hospital nurse educators (Baker et al. 2019; Nofal et al. 2018). Similarly, Öztekin et al. (2016) indicate a need for in-hospital disaster training programmes for nurses and that they learn more about disaster nursing. However, nurses with prior disaster experience have shown to be more confident in managing a disaster, as Jang and Cho (2022) identified in their cross-sectional study that compared to colleagues without disaster knowledge, rural nurses with prior disaster nursing education had showcased higher disaster nursing competencies.

Wenji et al. (2015) recommended the need to develop and find effective plans, research and education about disaster responses, as well as include military nurses in policy development of disaster response. To ensure that nurses are adequately disaster prepared, Usher et al. (2015) recommended the future development of disaster policy. Furthermore, Labrague et al. (2018) brought across a recommendation for disaster management protocols to be developed and formulated.

A disaster is an event that occurs unexpectedly, and its preparation is, therefore, essential. To aid in the preparation processes, hospital disaster policies, protocols and guidelines are requisite to prevent mitigating factors and promote competency in nurses who work in the emergency department. Furthermore, these plans inform emergency nurses of roles they are to assume should this event occur. Supported by Al Thobaity et al. (2015), a need for nursing education in disaster management and education and training in nurses’ roles during the response to a disaster was identified. A recommendation for hospital nurses to formulate a curriculum that would focus on promoting disaster preparedness, disaster management plans and nurses’ roles was made by Baker et al. (2019).

Schultz et al. (2012) identified a lack of time and space to accommodate expanded disaster curricula in the current programme, a lack of time or limited time to attend training by professionals and multiple training courses outside disaster medicine, among other barriers. However, instructional strategies on courses of curricula developed such as blended learning, distance learning, integration of realistic scenarios in training, application of active learning and teaching acute care physicians, nurses and emergency medical services (EMS) are recommended by Schultz et al. (2012) to teach content that aligns with the performance objectives and to furthermore to meet the training objectives. To understand effective strategies required for disaster preparedness education and training for emergency nurses, Hammad et al. (2017) recommend that research be undertaken to improve nurses’ understanding of effective strategies to prepare nurses for disaster response. This will explore further factors that would make emergency nurses disaster-ready and confident. Attending disaster preparedness education and training, workshops and training programmes has been identified as necessary in improving emergency nurses’ disaster preparedness and confidence in managing such. Providing disaster training that ranges from didactic lectures and disaster drills by healthcare authorities and universities, as well as training programmes and disaster drills, including a mix of tabletop exercises, is recommended (Taskiran & Baykal 2019).

### Limitations

Most of the articles were published in English; however, a small percentage were in other languages not known to the researcher; from the title search results, it was identified that such could have been potentially included in the study.

### Implications and recommendations

Emergency nurses’ disaster competencies are essential for disaster preparation, response and mitigation. The review highlighted some key competencies that need to be emphasised during disaster preparation and management for emergency nurses. For nurses to be adequately prepared for disasters, further education and training such as simulation, drills and tabletop exercises based on the identified competencies is essential and thus recommended. Furthermore, support from nursing managers and leaders is required to create training opportunities and policies that will encourage and enhance disaster preparedness for this calibre of nurses.

## Conclusion

This FMRS outlined the most common competencies that are important for emergency nurses’ disaster preparation and management. Integrating these competencies in disaster plans, policies and procedures, as well as continuous education and training of emergency nurses, will grant nurses confidence in preparing for and managing a disaster occurrence.
